# Fungal communities in bat guano, speleothem surfaces, and cavern water in Madai cave, Northern Borneo (Malaysia)

**DOI:** 10.1080/21501203.2021.1877204

**Published:** 2021-01-25

**Authors:** Ibrahem G. Wasti, Faisal Ali Anwarali Khan, Henry Bernard, Noor Haliza Hassan, Tom Fayle, Jaya Seelan Sathiya Seelan

**Affiliations:** a Institute for Tropical Biology and Conservation, Universiti Malaysia Sabah, Kota Kinabalu, Sabah, Malaysia;; b Faculty of Natural Science and Sustainability, University College Sabah Foundation, Sabah, Malaysia; c Department of Zoology, Faculty of Resource Science and Technology, Universiti Malaysia Sarawak, Sarawak, Malaysia; d Department of Biodiversity and Conservation Biology, Biology Centre, Czech Academy of Sciences, Ceske Budejovice, Czech Republic

**Keywords:** Biodiversity, borneo, fungi, limestone cave, Madai

## Abstract

The island of Borneo is a global biodiversity hotspot. However, its limestone caves are one of its least-studied ecosystems. We report for the first time the fungal species richness, diversity and abundance from Madai cave, situated in north-eastern Borneo. Environmental samples from inside the cave environment were collected (guano, speleothem, and cavern water) via opportunistic sampling. The dilution method was performed for isolation of fungi. Morphological characterisation and molecular analysis of the ITS region were utilised for the identification of isolates. Fifty-five pure cultures of fungi were attained, comprising 32 species from 15 genera, eight orders, and two divisions, Ascomycota and Basidiomycota. Ascomycetes dominated the fungal composition, accounting for 53 (96%) out of 55 total isolates. *Penicillium* spp. accounted for more than 47.1% of fungal abundance in all sample types. However, *Aspergillus* spp. had the highest occurrence rate, being isolated from all environmental samples except one. *Purpureocillium lilacinum* was isolated most frequently, appearing in five separate samples across all three substrates. *Annulohypoxylon nitens, Ganoderma australe, Pyrrhoderma noxium*, and *Xylaria feejeensis* were discovered and reported for the first time from the cave environment. This study provides additional data for further research on the mycoflora of Sabah’s various ecosystems, especially limestone caves.

## Introduction

Caves are unique ecosystems that are relatively underexplored in Borneo. This is particularly true for its microorganisms, especially the diversity of fungi in cave environments. In contrast to the external environment, caves are dark, relatively cool, humid, and nutrient-limited (Gabriel and Northup 2013; Zhang et al. 2017, 2018). The lack of photosynthetic organisms influences the oligotrophic nature of the cave, which in turn influences its mycofloral diversity (Gunde-Cimerman et al. 1998; Hose et al. 2000; Barton and Jurado 2007; Kuzmina et al. 2012; Gabriel and Northup 2013; Ogórek et al. 2013). Fungi are some of the most dominant organisms in caves due to the high rate of spore dissemination, colonisation capability in various substrates, and tolerance to a wide range of pH values (Nováková 2009; Bastian et al. 2010; Wang et al. 2010; Ogórek et al. 2013). Over 1150 species of fungi have been recorded from caves throughout the world, with the most species-rich division being Ascomycota, followed by Basidiomycota and Zygomycota with fewer species (Vanderwolf et al. 2013). Many of the fungi found in cave systems are not native to caves but are likely introduced and dispersed by humans, fauna, water sources, and air currents (Jablonsky et al. 1993; Ikner et al. 2007; Shapiro and Pringle 2010; Vanderwolf et al. 2016; Nováková et al. 2018). Some suspected obligate troglobitic fungi have been reported, such as *Acaulium caviariforme* (Vanderwolf et al. 2013), *A. baecitus* (Nováková et al. 2012), and *A. thesauricus* (Nováková et al. 2012). However, the existence of true obligate troglobitic fungi remains contentious. It is estimated that only 3–8% of all fungi on earth have been identified and described, and an overwhelming majority of extant fungi remain to be discovered (Hawksworth and Lücking 2017). Furthermore, the cave-dwelling fungi of the tropics, and of Malaysian Borneo in particular, remains very poorly documented.

Fungi play vital roles in the ecosystems they inhabit, whether as saprophytes, symbionts, parasites, or food sources (Bastian et al. 2010; Araújo and Hughes 2016). Mycoses are rapidly becoming one of the leading threats to wildlife with numerous epidemics around the world, especially in tropical regions due to their warm, humid climates (Jurado et al. 2010; Fisher et al. 2012; Hsu et al. 2012). White-nose Syndrome (WNS) (Puechmaille et al. 2011), chytridiomycosis (Fisher et al. 2009) and snake fungal disease (Lorch et al. 2016) are all severe and often fatal fungal disease towards their host animal populations. However, of these diseases, only WNS is found exclusively in caves.

Cave fungi have been isolated from various substrates, including sediment, wall, speleothem, guano, water, air, and various fauna (Jurado et al. 2008; Vanderwolf et al. 2013). While cave fungal studies specific to speleothem fungi remain scant, studies on the relationship of fungi with cave walls and overall cave morphology have been conducted. Endolithic growth of lithobiontic fungi can biologically weather rock surfaces, but in the long term they can help stabilise and preserve rock surface morphology (Hoppert et al. 2004). Lithogenic fungi can be detrimental to anthropological sites, especially due to cave wall staining of ancient paintings (Bastian et al. 2010). Cavern water indirectly affects cave mycoflora in various ways (Cunningham et al. 1995; Vanderwolf et al. 2013). Non-native fungal species and organic material may be introduced into the cave via running water through the cave floor or vertical filtration of rainwater from the soil above (Dupont et al. 2007; Ikner et al. 2007). The moist microclimate, stable temperatures, and abundance of nutrients of guano make it one of the most dominant fungal substrates in caves, effectively serving as a separate micro-ecosystem (Paulson 1972; Nieves-Rivera et al. 2009). In Domica cave, Slovakia, guano had the greatest fungal diversity when compared to eight other substrate types (Nováková 2009).

Ecological factors that affect levels of fungal diversity in caves include seasonal changes, temperature, humidity, rainfall, human activity, sunlight, and distance from cave entrance (Wang et al. 2010; Taylor et al. 2014). Furthermore, the frequency of human visitation and other anthropogenic influences may affect the cave mycoflora. Increased human traffic into cave systems can cause contamination of indigenous fungal communities by repeated introductions of non-indigenous microorganisms and nutrients (Ikner et al. 2007; Chelius et al. 2009; Porca et al. 2011; Griffin et al. 2014). Shapiro and Pringle (2010) reported that increased human visitation is correlated with lower levels of fungal diversity in caves. Human disturbance in caves is usually localised to the specific areas of interaction. However, increased human visitation is correlated with increased fungal abundance (Wang et al. 2010; Porca et al. 2011). Regular visits by humans allow for certain fungi to survive in contaminated areas because visitors tend to leave behind food waste and other organic materials (Ogórek et al. 2013; Griffin et al. 2014). Even fungi native to the cave could be dispersed to other areas of the cave they did not occupy before human disturbance.

Subterranean ecosystems are inhabited by organisms that have adapted to tolerate relatively unfavourable and niche conditions, including fungi (Ogórek et al. 2017. Madai cave is known to have accommodated early humans based on excavation of faunal remains (Harrison 1998). A variety of bats roost in the cave, with some colonies having populations over 100,000 individuals (Kobayasi et al. 1980). These bats bring in nutrient sources from outside the cave daily, and their guano and cadavers are known to harbour fungi (Nieves-Rivera et al. 2009; Nováková et al. 2018). Madai cave is also subject to major anthropogenic influence due to land use for palm-oil plantations surrounding the forest reserve, seasonal swiftlet farming harvesting by the local community for generations, and visits by tourists from all over the world as a major eco-tourism attraction. The aim of this study was to establish baseline data that will determine the fungal diversity existing in Sabah’s caves and its ecological relationships. This study is the first of its kind in Madai cave, as most studies have been archaeological or faunal. Better understanding of the ecological roles and interactions of fungi and reporting on the potential existence of pathogenic fungi will allow improved management practices to cave caretakers and stakeholders, especially with the influx of tourists and professionals that often visit cave areas in Sabah.

## Materials and methods

### Site description

**Figure 1.**
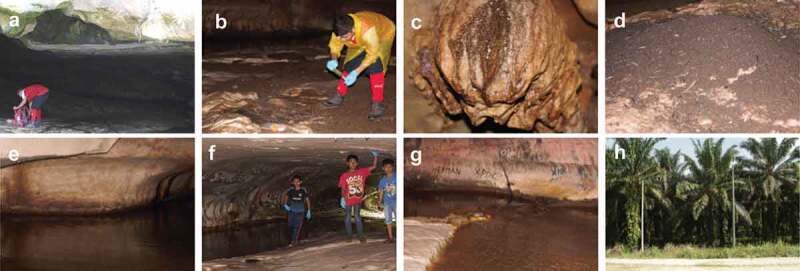
Madai Cave. A. Cave entrance. B. Researcher collecting guano sample. C. Speleothem. D. Guano pile. E. Cave stream deep in the cave. F. Village children playing near the cave entrance. G. Visible graffiti on cave wall. H. Land use for palm oil surrounding Baturong Madai Forest Reserve

Madai Cave, Baturong Madai Forest Reserve, Class VI (Virgin Forest), Kunak, Sabah (4°41ʹ10.01”N 118°15ʹ4.12”E) were visited on 28–29 November 2017. A small village is located immediately outside the cave entrance, and there are two main chambers of the cave. The first one has an entrance at the ground level, and it is in this lower chamber that the sampling for this study took place (Figure 1A-G). A second chamber is located about a 100 m hike up the limestone formation, past some ancient burial sites. The air temperature in the cave on the day of sampling fluctuated from 27°C and rose to around 29°C near the cave entrance. The air humidity ranged between 92% and 100%. The main chamber of the cave is more than 400 m in length, not including the multiple branches of the main chamber (Wilford 1961). In this study, only the first 100 m of the main chamber was sampled and explored.

The cave is open to the public during the off seasons of swiftlet nest harvesting and is often visited by large groups of both foreigners and locals. Immediately outside of the forest reserve exist monocrop farms for palm oil production (Figure 1H), which is what most of the unprotected forest areas have been converted into in this region.

For the purposes of this study, the cave was categorised into three separate zones based on light intensity. From the entrance, the first 20 m of the cave was designated as the lighted zone due to its exposure to direct sunlight. The twilight zone exists around 20–40 m within the cave, and it is defined as dimly lit areas that are not exposed to direct sunlight. Past the twilight zone area, the remainder of the cave is pitch black since there are no apertures to allow natural light into the cave. These unlighted areas are labelled as the dark zone of the cave, and it can only be properly traversed by humans if artificial light sources are available.

### Sampling and identification

**Figure 2.**
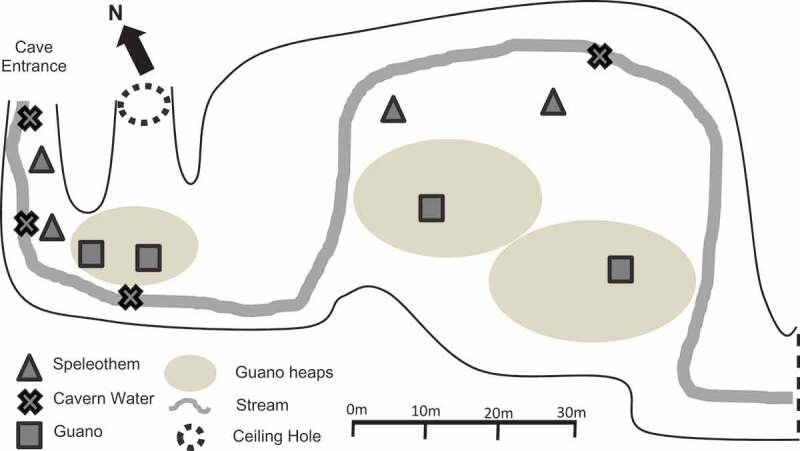
Layout of the first 100 m of Madai Cave with sampling sites

Opportunistic sampling of speleothem, cavern water, and guano was utilised, in which four samples of each substrate were acquired from the first 100 m from the cave entrance (Figure 2). Speleothem was sampled using the swab method (25 cm^2^ area per swab) and stored in sterile sample tubes, capped, and sealed (Ikner et al. 2007; Vaughan et al. 2011). Guano samples (10 g) were collected using sterile scoops and stored in sterile sample tubes (Nieves-Rivera 2003). Samples of cavern water (10 ml) were also collected and sealed in sterile sample tubes. The distance from entrance was recorded for all collections immediately after sealing. Samples were labelled with a letter designating substrate type and a number in order of increasing distance from entrance (*e.g*. speleothem sample 1 = S1; speleothem samples 2 = S2). All samples were collected in triplicate and chilled in ice (<4°C) until transported to the mycology laboratory in the Institute for Tropical Biodiversity and Conservation, Universiti Malaysia Sabah. In the laboratory, samples were immediately stored in a chiller at <4°C until isolation.

Samples were serially diluted 10-fold up to 10^−4^, and 1 ml aliquots of the 1 x 10^−2^ 1 x 10^−3^, and 1 x 10^−4^ dilutions were selected for plating. The samples were placed in Petri dishes via serial dilution onto Potato Dextrose Agar (PDA) and Malt Extract Agar (MEA) infused with 40 mg/L streptomycin sulphate, which was incubated from 7 to 28 days at 25 ± 1°C in the dark. Dilutions and plating were performed in triplicate. The colonies that appeared on the media were categorised by Morphological Taxonomic Units (MTU) and was counted. Pure isolates were obtained using the three–point method on PDA and MEA before morphological and genetic analysis. All pure isolates underwent morphological identification based on universal identification keys described by Raper and Fennel (1965), Domsch et al. (1980), and Klich (2002).

DNA from pure cultures (7–14 days old) was extracted using the E.Z.N.A. DNA Fungal Kit (Omega Bio-Tek, USA) following the manufacturer’s instructions after seven days of incubation. The internal transcribed spacer region of fungal rDNA was amplified using the primers ITS1 (5ʹ-TCCGTAGGTGAACCTGCGG-3ʹ) and ITS4 (5ʹ-TCCTCCGCTTATTGATATGC-3ʹ) (White et al. 1990). Polymerase chain reaction (PCR) amplifications were performed in a Bio-Rad T100 Thermal Cycler according to Ogórek et al. (2019). The amplification products were electrophoresed in a 1% agarose gel for 30 mins, which was stained with gel red for visualisation. The PCR products were purified using Column-Pure PCR Clean-Up Kit (Applied Biological Materials, Inc.) according to manufacturer protocols and sequenced by MyTACG Bioscience SDN. BHD. (Kuala Lumpur, Malaysia).

### Data analysis

The ITS sequences were assembled by BioEdit Sequence Alignment Editor version 7.2.5. The top hit sequences were determined by comparing the obtained sequences with those deposited in the GenBank database using the BLASTn algorithm (http://www.ncbi.nlm.nih.gov). The fungal abundance data obtained from the serial dilution of fungal colonies cultured in Petri dishes were expressed as colony-forming units (CFU). This was calculated with the formula X = (a x p)/V, where “a” is the number of colonies, “V” is the inoculation aliquot volume, and “p” is the dilution factor. The final CFU count for each sample recorded is the average of nine dilution plates per sample. The fungal abundance data were then used for diversity and evenness analysis in PAST 3.10 software. The diversity analyses were run separately for each substrate type due to difference in units of abundance (*i.e*. CFU/cm^2^, CFU/ml, and CFU/g). Fungal species occurrence is defined as the number of times the same species was isolated as pure culture (maximum of once per sample).

## Results

**Figure 3.**
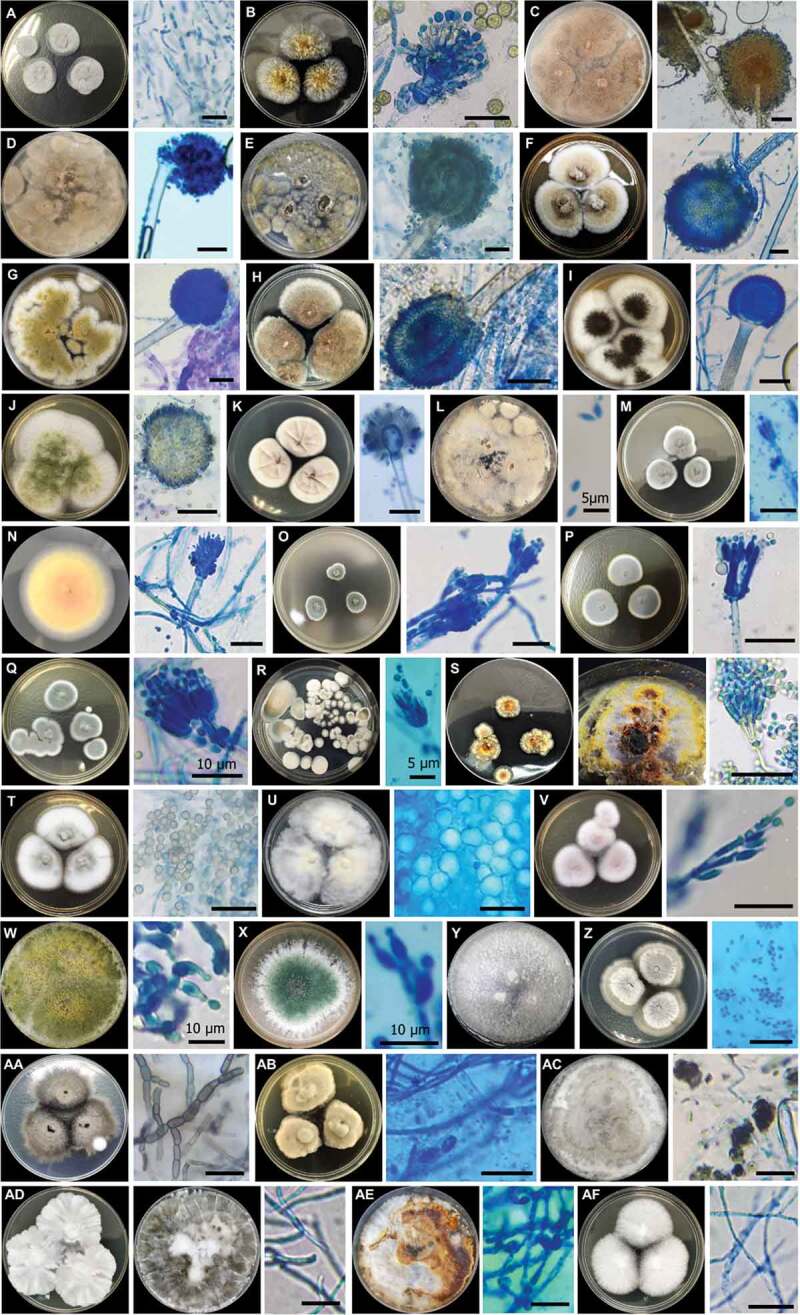
The relative abundance of genera isolated from Madai Cave in different substrates

**Figure 4.**
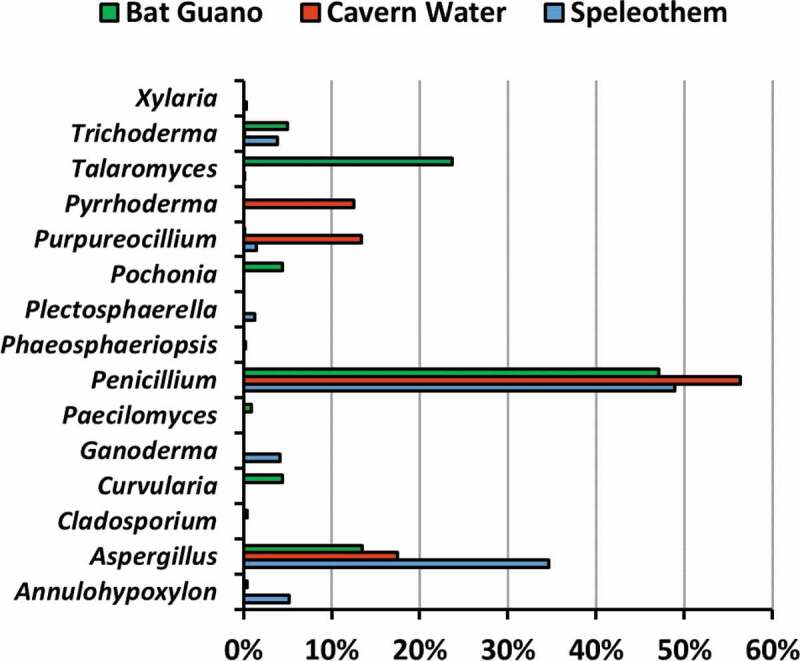
Culturable fungi of Madai Cave, Sabah, 7–14 day-old cultures at 25°C, top view of colony and micromorphology under microscope on PDA media. A. *C. cladosprioides*. B. *Aspergillus* sp. 1. C. *Aspergillus* sp. 2. D. *Aspergillus* sp. 3. E. *A. aculeatus*. F. *A. europaeus*. G. *A. flavus*. H. *A. japonicus*. I. *A. niger*. J. *A. nomius*. K. *A. sydowii*. L. *P. variotii*. M. *Penicillium* sp. 1. N. *Penicillium* sp. 2. O. *P. bilaiae*. P. *P. citrinum*. Q. *P. paxilli*. R. *P. simplicissimum*. S. *Talaromyces* sp. T. *T. minioluteus*. U. *P. chlamydosporia*. V. *P. lilacinum*. W. *T. asperellum*. X. *T. harzianum*. Y. *T. paraviridescens*. Z. *P. cucumerina*. AA. *C. lunata*. AB. *Phaeosphaeriopsis* sp. AC. *A. nitens*. AD. X. *feejeensis*. AE. *P. noxium*. AF. *G. australe*. Scale bars = 20 µm (unless otherwise labelled)

In total, 55 fungal isolates (pure cultures) were obtained. Twenty-three isolates were obtained from four speleothem samples, 15 isolates from four cavern water samples, and 17 isolates from four guano samples. The fungi were classified into 32 species 15 genera, eight orders, and two divisions, namely Ascomycota and Basidiomycota (Table 1), based on morphological characters (Figure 4) and molecular analysis of the ITS region for confirmation. Only two out of 32 species were identified as Basiodiomycota, *Ganoderma australe* and *Pyrrhoderma noxium*. Out of the 55 total isolates, 31 underwent DNA extraction (ITS) for subsequent molecular analysis to corroborate morphological identification and help identify cryptic taxa. All 31 extracted DNA samples were successfully sequenced, and BLASTn sequence analysis corroborated 24 out of 32 total species identified in this study (Table 2). All BLASTn results had identity matches over 96.7%, except for *Talaromyces* sp. (92.2%). The fungus that had the most frequent occurrence was *Purpureocillium lilacinum*, which was isolated from five different samples composed of all three substrate types. However, the most frequently isolated genus was *Aspergillus*, accounting for 36.4% of all isolates. Based on fungal abundance data, *Penicillium* spp. dominated fungal composition and accounted for 56.3%, 48.9%, and 47.1% of cavern water, speleothem and guano fungi, respectively (Figure 3).

**Table 1.** Fungal taxa cultured from madai cave, malaysia

	Substrate			Total Occurrence (n)
Fungi	Speleothem	Cavern Water	Guano	
ASCOMYCOTA				
Capnodiales				
*Cladosporium cladosporioides* (Fresen.) G.A. de Vries			1 D^1^	1
Eurotiales				
*Aspergillus* sp. 1	1 D		1 D	2
*Aspergillus* sp. 2	1 M^a^			1
*Aspergillus* sp. 3	1 M			1
*A. aculeatus* Iizuka	1 M	2 M	1 D	4
*A. europaeus* Hubka, A. Nováková, Samson, Houbraken, Frisvad, M. Kolařík	1 M			1
*A. flavus* Link	2 M	1 M		3
*A. japonicus* Saito		1 D, 1 M		2
*A. niger* Tiegh.	1 D		1 M	2
*A. nomius* Kurtzman, B.W. Horn & Hesselt.	1 D	1 D	1 D	3
*A. sydowii* (Bainier & Sartory) Thom & Church		1 D		1
*Paecilomyces variotii* Bainier			1 D, 1 M	2
*Penicillium* sp. 1		1 M		1
*Penicillium* sp. 2			1 M	1
*P. bilaiae* Chalab.	1 D			1
*P. citrinum* Thom	2 M	1 D		3
*P. paxilli* Bainier	1 M	1 M	1 M	3
*P. simplicissimum* (Oudem.) Thom			1 D	1
*Talaromyces* sp.	1 D			1
*T. miniluteus* (Dierckx) Samson, Yilmaz, Frisvad & Seifert			1 D	1
Hypocreales				
*Pochonia chlamydosporia* (Goddard) Zare & W. Gams			1 D	1
*Purpureocillium lilacinum* (Thom) Luangsa-ard, Houbraken, Hywel-Jones & Samson	2 D	2 D	1 M	5
*Trichoderma asperellum* Samuels, Lieckf. & Nirenberg	1 M		1 D	2
*T. harzianum* Rifai	2 M	1 M		3
*T. paraviridescens* Jaklitsch, Samuels & Voglmayr			1 D	1
*Incertae sedis*				
*Plectosphaerella cucumerina* (Lindf.) W. Gams	1 D			1
Pleosporales				
*Curvularia lunata* (Wakker) Boedijn			1 D	1
*Phaeosphaeriopsis* sp.		1 D		1
Xylariales				
*Annulohypoxylon nitens* (Ces.) Y.M. Ju, J.D. Rodgers, & H.M. Hsieh	1 D		1 D	2
*Xylaria feejeensis* (Berk.) Fr.	1 D			1
**BASIDIOMYCOTA**				
Hymenochaetales				
*Pyrrhoderma noxium* (Corner) L.W. Zhou & Y.C. Dai		1 D		1
Polyporales				
*Ganoderma australe* (Fr.) Pat.	1 D			1
**TOTAL**	**23**	**15**	**17**	**55**

^1^ = identified by DNA BLASTn (GenBank) and phylogenetic analysis, and corroborated by morphological characterisation.

^a^= identified by morphological characterisation only.

**Table 2.** Culturable cave fungi of madai cave, sabah BLASTn (genBank) analysis

**Fungal Taxa**	**Source Sample**	**NCBI Identification**	**Query Cover (%)**	**Identities (%)**	***E* value**	**NCBI** **Accession**
*Annulohypoxylon nitens*	G2	KU684021	92%	99.3%	0.0	MN783056
*Annulohypoxylon nitens*	S3	FN252415	98%	99.0%	0.0	MN783036
*Aspergillus* sp. 1	G4	MK638758	100%	96.9%	0.0	MN783044
*Aspergillus* sp. 1	S3	MH517369	100%	96.9%	0.0	MN783034
*Aspergillus aculeatus*	G2	MK280716	100%	100.0%	0.0	MN783053
*Aspergillus japonicus*	W4	KF800630	100%	100.0%	0.0	MN783040
*Aspergillus niger*	S3	MK203789	100%	100.0%	0.0	MN783035
*Aspergillus nomius*	G3	MH279416	99%	99.7%	0.0	MN783061
*Aspergillus nomius*	S4	MH279388	99%	99.8%	0.0	MN783075
*Aspergillus nomius*	W3	MH279387	100%	100.0%	0.0	MN783057
*Aspergillus sydowii*	W2	KX674612	100%	100.0%	0.0	MN783070
*Cladosporium cladosporioides*	G2	EF405864	99%	100.0%	0.0	MN783059
*Curvularia lunata*	G2	JN116704	93%	100.0%	0.0	MN783060
*Ganoderma australe*	S3	LC084692	94%	99.4%	0.0	MN783025
*Paecilomyces variotii*	G4	FJ345354	99%	100.0%	0.0	MN783021
*Penicillium bilaiae*	S3	LN901118	100%	96.7%	0.0	MN783041
*Penicillium citrinum*	W4	GU566273	99%	99.5%	0.0	MN783043
*Penicillium simplicissimum*	G2	HQ607866	99%	99.8%	0.0	MN783054
*Phaeosphaeriopsis* sp.	W4	KF800300	99%	99.5%	0.0	MN783087
*Plectosphaerella cucumerina*	S2	EU326201	97%	99.5%	0.0	MN783077
*Pochonia chlamydosporia*	G2	EU266591	97%	99.8%	0.0	MN783069
*Purpureocillium lilacinum*	S3	KY951911	100%	99.7%	0.0	MN783024
*Purpureocillium lilacinum*	S4	MH860675	99%	99.8%	0.0	MN783073
*Purpureocillium lilacinum*	W1	MH860675	100%	99.7%	0.0	MN783020
*Purpureocillium lilacinum*	W2	MH860675	99%	98.6%	0.0	MN783058
*Pyrrhoderma noxium*	W2	KU194338	99%	98.5%	0.0	MN783062
*Talaromyces* sp.	S3	MH857785	99%	92.2%	0.0	MN783022
*Talaromyces minioluteus*	G2	MH857785	100%	99.5%	0.0	MN783055
*Trichoderma asperellum*	G4	KY623504	99%	100.0%	0.0	MN783026
*Trichoderma paraviridescens*	G2	MF782827	99%	99.8%	0.0	MN783049
*Xylaria feejeensis*	S3	KY951907	99%	99.7%	0.0	MN783023

In speleothem samples, the average fungal abundance was 229.3 CFU/cm^2^. The isolate with highest abundance count from speleothem was the *Penicillium citrinum* from sample S1 (272.4 CFU/cm^2^). The average fungal abundance of cavern water samples was 335.0 CFU/ml, and *Penicillium* sp. 1 from sample W1 had the highest abundance (716.7 CFU/ml). In guano samples, the average fungal abundance was 6,266.7 CFU/g, and the single isolate which had the highest abundance count was *Penicillium paxilli* from sample G1 (8,922.2 CFU/g) (Table 3). Both Shannon-Wiener and Simpson alpha diversity indices showed that speleothem samples had the most diverse fungal communities, followed by guano, and then cavern water (Table 4). Additionally, fungal abundance in speleothem seemed to decrease with increasing distance from the cave entrance, although there were only four samples with abundance of data for speleothem in this study.

**Table 3.** Average abundance of culturable fungi of madai cave, sabah (CFU per unit sample)

Sample (Distance from Entrance)	Fungal Taxa	Identification	CFU/unit^a^	
			Fungal Taxa	Sample Total
Speleothem				x = 229.3
S1 (1 m)	*Aspergillus* sp. 3	Morphology	72.9	554.7
	*Aspergillus flavus*	Morphology	222.7	
	*Penicillium paxilli*	Morphology	259.1	
S2 (16 m)	*Aspergillus aculeatus*	Morphology	4.4	381.3
	*Aspergillus flavus*	Morphology	44.9	
	*Penicillium citrinum*	Morphology	272.4	
	*Plectosphaerella cucumerina*	DNA^a^	15.1	
	*Trichoderma harzianum*	Morphology	44.4	
S3 (55 m)	*Annulohypoxylon nitens*	DNA	62.7	175.1
	*Aspergillus* sp. 1	DNA	1.8	
	*Aspergillus europaeus*	Morphology	4.4	
	*Aspergillu niger*	DNA	0.9	
	*Ganoderma australe*	DNA	49.8	
	*Penicillium bilaiae*	DNA	44.4	
	*Purpureocillium lilacinum*	DNA	4.9	
	*Talaromyces* sp.	DNA	0.4	
	*Trichoderma asperellum*	Morphology	1.3	
	*Xylaria feejeensis*	DNA	4.4	
S4 (75 m)	*Aspergillus* sp. 2	Morphology	13.3	86.2
	*Aspergillus nomius*	DNA	49.3	
	*Penicillium citrinum*	Morphology	9.3	
	*Purpureocillium lilacinum*	DNA	13.3	
	*Trichoderma harzianum*	Morphology	0.9	
Cavern Water				x = 335.0
W1 (3 m)	*Aspergillus japonicus*	Morphology	25.6	744.4
	*Penicillium* sp. 1	Morphology	716.7	
	*Purpureocillium lilacinum*	DNA	2.2	
W2 (17 m)	*Aspergillus aculeatus*	Morphology	3.3	488.9
	*Aspergillus flavus*	Morphology	17.8	
	*Aspergillus sydowii*	DNA	123.3	
	*Purpureocillium lilacinum*	DNA	176.7	
	*Pyrrhoderma noxium*	DNA	167.8	
W3 (30 m)	*Aspergillus aculeatus*	Morphology	36.7	81.1
	*Aspergillus nomius*	DNA	13.3	
	*Penicillium paxilli*	Morphology	30.0	
	*Trichoderma harzianum*	Morphology	1.1	
W4 (85 m)	*Aspergillus japonicus*	DNA	14.4	25.6
	*Penicillium citrinum*	DNA	7.8	
	*Phaeosphaeriopsis* sp.	DNA	3.3	
Guano				x = 6266.7
G1 (26 m)	*Paecilomyces variotii*	Morphology	33.3	8,955.6
	*Penicillium paxilli*	Morphology	8,922.2	
G2 (30 m)	*Annuhypoxylon nitens*	DNA	111.1	13,622.2
	*Aspergillus aculeatus*	DNA	1,122.2	
	*Cladosporium cladosporioides*	DNA	111.1	
	*Curvularia lunata*	DNA	1,111.1	
	*Penicillium simplicissimum*	DNA	2,877.8	
	*Pochonia chlamydosporia*	DNA	1,111.1	
	*Talaromyces minioluteus*	DNA	5,944.4	
	*Trichoderma paraviridescens*	DNA	1,233.3	
G3 (53 m)	*Aspergillus nomius*	DNA	1,111.1	1,111.1
G4 (75 m)	*Aspergillus* sp. 1	DNA	1,122.2	1,377.8
	*Aspergillus niger*	Morphology	22.2	
	*Paecilomyces variotii*	DNA	200.0	
	*Penicillium* sp. 2	Morphology	11.1	
	*Purpureocillium lilacinum*	Morphology	11.1	
	*Trichoderma asperellum*	DNA	11.1	

^a^unit = *CFU/cm^2^* for speleothem samples, *CFU/ml* for cavern water samples, and *CFU/g* for guano samples.

^a^All isolates identified by molecular characterisation is also corroborated by morphological characterisation.

**Table 4.** Diversity index scores based on abundance counts of all taxa isolated

**Cave**	**Sample**	**Simpson** (1-D)	**Shannon-Wiener** (*H*)	**Evenness** (e*^H^*/*S*)
Madai	Guano	0.79	1.91	0.42
	Speleothem	0.84	2.15	0.45
	Cavern water	0.67	1.53	0.39

## Discussion

This is the first study on fungi in Madai cave. Studies in limestone caves in Sabah have been limited to the exploration of their fauna, geology, or anthropogenic value, and fungi have often been overlooked. Thirty-four (61.8%) fungal isolates in this study were of the order Eurotiales, including *Aspergillus* spp. (36.4%), *Penicillium* spp. (18.2%), *Talaromyces* spp. (3.6%), and *Paecilomyces variotii* (3.6%). All isolates cultured were Ascomycota except for two Basidiomycota isolates. Fungi in the genera *Penicillium* and *Aspergillus* represented most abundant taxa for all substrates. Our results reflect those from previous fungal studies from cave ecosystems as Ascomycota fungi usually dominate the fungal composition of cave ecosystems, followed by Basidiomycota, Zygomycota, and then others (Vanderwolf et al. 2013). Four species of fungi were discovered from cave samples for the first time in this study, namely *A. nitens, Ganoderma australe, Pyrrhoderma noxium*, and *Xylaria feejeensis* (Vanderwolf et al. 2013; Nováková et al. 2018; Karunarathna et al. 2020; Zhang et al. 2020; Cunha et al. 2020).

Prior to the study, guano was hypothesised to have the largest diversity compared to speleothem surfaces and cavern water (Nováková 2009; Vanderwolf et al. 2013). Instead, the results showed that speleothem surfaces had the highest diversity indices (1-D = 0.84, H = 2.15), most pure isolates attained (23), and highest number of taxa identified (19). Speleothem surfaces yielded the highest proportion of isolates (41.8%), which included 19 species from nine genera and four orders. Only one isolate from speleothem was a basidiomycete (*Ganoderma australe*) and all others were ascomycetes. Fungi have been isolated from cave walls and sediment in previous studies, and fungi are postulated to participate in speleothem deposition (Engel et al. 2004; Bastian et al. 2010). Our results are congruent with a previous study on cave fungi, where *Aspergillus* and *Penicillium* fungi dominated the speleothem fungal community (Vaughan et al. 2011).

Fungi have been isolated from cave walls and sediment in previous studies, and these fungi are postulated to participate in speleothem deposition and biomineralization (Northup and Lavoie 2001; Engel et al. 2004; Barton and Northup 2007; Bastian et al. 2010). The speleothem in Madai cave, particularly those sampled, was wet due to vertical filtration of water from above. Wet, mouldy speleothem is an indication that they are biologically active (Dodge-Wan and Deng 2013). Fungal richness is often associated with substrate moisture (Schimel et al. 1999; Frey et al. 1999). Additionally, fungal spores have been shown to colonise and grow on virtually rock surface that has even minute traces of carbon (Wainwright et al. 1993; Barton and Jurado 2007). Vanderwolf et al. (2013) showed in her world review that cave sediment (43%) and cave wall (18%) both individually composed of a higher percentage of fungal taxa than guano (16%). Although this is not speleothem, it does show that fungal diversity on cave rocks seems to be higher than that of guano.

Distance from cave entrance may affect fungal abundance on speleothem surfaces. This result concurs with previous studies that measured similar data, as fungal biodiversity, species occurrence, abundance, and biomass decreases with distance from cave entrance (Hsu and Agoramoorthy 2001; Kuzmina et al. 2012; Mulec et al. 2012; Taylor et al. 2014). There are a multitude of factors that may contribute to this relationship. Primarily, many of the fungi found in caves originate from the external environment and is introduced by a multitude of methods, such as air currents, humans, and fauna (Shapiro and Pringle 2010; Porca et al. 2011; Ogórek et al. 2013; Pusz et al. 2014, 2015; Vanderwolf et al. 2016). However, Zhang and Cai (2019) reported that distance did not play a role in species richness. Instead, they showed that similarities of fungal communities inside and outside the cave decreased with increasing distance from the cave entrance. Since cosmopolitan soil fungi tend to be isolated at higher rates using culture-dependent methods, it may reflect on the results of this study and prior studies that rely on culture-based isolation. Nonetheless, if a cave has speleothem formations anywhere within the cave site, it should be assumed that fungi are active and present on these formations.

Guano yielded the second most isolates in this study (30.1%). A total of 16 species from 11 genera and 5 orders were identified from this substrate, and all of them were ascomycetes. Oligotrophy is a major limiting factor for fungi in caves, thus higher fungal diversity will likely be found on substrates with higher organic concentrations (Bastian et al. 2010; Jurado et al. 2010; Kuzmina et al. 2012). Guano is one of the major sources of organic matter in caves, and a broad spectrum of microfungal species are usually isolated from guano, including pathogenic yeasts, basidiomycetous yeasts, keratinophilic fungi, and ubiquitous ascomycetes (Larcher et al. 2003; Nováková 2009). Any cave that serves as bat roosts and is littered in one form or another with guano is likely to be reservoirs to a wide array for fungi.

Cavern water had the lowest proportion of fungal isolates compared to the other two substrates in this study (27.3%). A total of 12 species from six genera and four orders were identified from cavern water. Only one isolate was identified as a basidiomycete (*Pyrrhoderma noxium*), and the remaining are all ascomycetes. The water samples from this study came from a singular, minimally branched stream that ran through the cave towards the mouth of the cave. Man et al. (2018) reported cavern water to contain the highest OTU count compared to other tested substrates, namely soil, rock, and air in caves. This is not the case in this study, but we sampled different substrates for comparison. Fungal dispersion and spore transmission via stream water are plausible explanations for our results. Fungi are expected to be isolated from cavern water because one of the main routes of organic matter transmission in caves is water flow (Ikner et al. 2007). Water flowing in and out of the cave serves as possible explanation for the transport and proliferation of fungi that can be isolated from water samples (Barton and Jurado 2007; Ortiz et al. 2014), especially since one of the main routes for organic matter to enter caves as dissolved organic carbon and colloidal organic matter through water flow (Ikner et al. 2007).

Some of the fungi identified in this study are known human pathogens. *Aspergillus* fungi are the most frequent cause of invasive mould infections in immunocompromised patients (Beck-Sagué and Jarvis 1993). While *A. fumigatus*, the most frequent cause of aspergillosis, was not reported in Madai cave, *A. flavus, A. nomius*, and *A. niger* can cause infection in humans and were isolated in this study (Marr et al. 2002; Caira et al. 2012). *Paecilomyces variotii* is another opportunistic human pathogen isolated from Madai cave, especially of immunocompromised patients (Sterflinger et al. 1999. *Curvularia lunata* is an opportunistic pathogen to humans and other animals, often infecting immunocompromised patients (Berman 2019). Any visitors or tourists entering in tropical caves should we aware of any potential risks posed by entering these ecosystems, especially those who may be immunocompromised.

There were several endophytic and phytopathogenic fungi isolated from Madai cave despite not having any autotrophs within the cave due to the lack of sunlight. Anthropogenic activity may explain how non-indigenous plant-associated fungi can be transported into novel ecosystems such as caves (Ikner et al. 2007; Shapiro and Pringle 2010). In Taiwan, distribution of *Pyrrhoderma noxium* is limited to areas with human activity, as no brown root rot has ever been found in undisturbed natural forests in Taiwan (Ann et al. 2002). Another possible method of introducing plant-associated fungi into the cave system is via arthropod vectors. The ability of some fungi to act as pathogens to both motile (fauna) and non-motile (flora) hosts could explain the role of arthropods as dispersers of endophytic and phytopathogenic fungi in caves. *Annulohypoxylon nitens* was isolated in this study. *Annulohypoxylon* fungi are associated with dead dicotyledonous wood as saprobes, but they are also found as endophytes and help promote growth in seed plants (Ju et al. 2017; Pereira et al. 2010; Ikeda et al. 2014). *Penicillium bilaiae* is another plant growth-promoting organism that was isolated in this study, as it has the ability to increase phosphorous nutrition in plants like wheat, medick, and lentil (Wakelin et al. 2007). On the other hand, phytopathogenic fungi identified in this study include *Curvularia lunata* (Liu et al. 2010), *Phaeosphaeriopsis* sp. (Golzar and Wang 2012; Thambugala et al. 2014), *Talaromyces minioluteus* (Stošić et al. 2019), *P. noxium* (Ann et al. 2002; Sahashi et al. 2014; Chung et al. 2015), and *Plectosphaerella cucumerina* (Carlucci et al. 2012; Li et al. 2017).

A number of fungi identified in this study are known as entomopathogens, many of which are being studied for their biological control potential. Madai cave is host to various invertebrates and acts as a roosting site for volant fauna such as bats and swiftlets. Animals are known to harbour fungi, and they are likely disseminators of fungal spores within caves, either as hosts, vectors, or cadavers (Vanderwolf et al. 2013; Nováková et al. 2018). *Penicillium citrinum* has been shown to cause mortality and reduced survival in *Culex quinquefasciatus* (mosquito) larvae after ingestion by the larvae (Maketon et al. 2013). *Plectosphaerella cucumerina* (Atkins et al. 2003), *Pochonia chlamydosporia* (Kerry 2000), and *Purpureocillium lilacinum* (Kepenekçi et al. 2018) are all being used and developed as biological control agents against plant pathogenic nematodes. *Trichoderma asperellum* and *T. harzianum* are both used as biological control agents against many plant disease-causing organisms, including *Phytophthora megakarya* (Deberdt et al. 2008), fungi (Watanabe et al. 2005; Alvindia and Hirooka 2011), and nematodes (Sharon et al. 2007). Insects feed on the fungi and bacteria that inhabit guano piles, which suggest their influence on the fungal community in caves both as consumers and dispersers (Šustr et al. 2005; Smrž et al. 2015). Arthropods are likely disseminators of cave fungi as many fungi isolated from cave environmental samples have been shown to include entomophilous, entomogenous, or entomopathogenic species (Ogórek et al. 2013; Vanderwolf et al. 2016).

This study on Madai cave’s fungal community serves to present baseline data with the purpose of serving a platform for future research of tropical mycota. Many fungi, especially microfungi, can only be identified with confidence to the genus level when using morphological analysis or when only utilising a single gene marker in phylogenetic analysis (Schoch et al. 2012). In this study, the utilisation of a combination of traditional morphological characterisation and molecular analysis allowed us to identify many of our specimens to the species level. However, our study only utilised culture-dependent methods of fungal isolation. Culture-dependent methods are known to only reveal as little as 0.6% to 8.0% of the total fungal species in a sample (Hibbett et al. 2009; Hawksworth and Lücking 2017). For a better understanding of cave mycobiota in Sabah, future studies should employ both morphological and molecular characterisation by implementing community-based culture-independent studies. Culture-independent methods, such as metagenomics and metabarcoding, can generate up to millions of raw sequences from a single sample and help eliminate bias towards fast-growing cosmopolitan fungi (Wiseschart et al. 2019; Zhang and Cai 2019). Hitherto, due to the lack of research on microfungi in Borneo, we are unaware of any deleterious fungal diseases in Sabah’s limestone caves that might infect fauna and humans.

Ongoing studies on fungi from various caves in Sabah are currently in progress. We urge more mycological studies and surveys to be conducted in caves in this region, not only to better understand fungal ecology, but to discover their enormous biological and industrial potential.

## Wordcount

6,408 words.
